# Potential Use of Plasma Rich in Growth Factors in Age-Related Macular Degeneration: Evidence from a Mouse Model

**DOI:** 10.3390/medicina60122036

**Published:** 2024-12-10

**Authors:** Eduardo Anitua, Francisco Muruzabal, Sergio Recalde, Patricia Fernandez-Robredo, Mohammad Hamdan Alkhraisat

**Affiliations:** 1 BTI Biotechnology Institute, 01005 Vitoria, Spain; kikomuru@gmail.com (F.M.); mohammad.hamdan@bti-implant.es (M.H.A.); 2 University Institute for Regenerative Medicine and Oral Implantology—UIRMI (UPV/EHU-Fundación Eduardo Anitua), 01007 Vitoria, Spain; 3 Experimental Ophthalmology Laboratory, Department of Ophthalmology, Clinica Universidad de Navarra, 31008 Pamplona, Spain; srecalde@unav.es (S.R.); pfrobredo@unav.es (P.F.-R.); 4 Navarra Institute for Health Research, IdiSNA, 31008 Pamplona, Spain; 5 Department of Oral and Maxillofacial Surgery, Oral Medicine and Periodontology Faculty of Dentistry, University of Jordan, Amman 11942, Jordan

**Keywords:** age-related macular degeneration, geographic atrophy, plasma rich in growth factors, retina, retinal pigment epithelial cell, platelet rich plasma (PRP)

## Abstract

*Background and Objectives*: Age-related macular degeneration (AMD) is the leading cause of low vision and legal blindness in adults in developed countries. Wet AMD can be successfully treated using vascular endothelial growth factor (VEGF) inhibitors; however, dry AMD currently has no effective treatment. The purpose of this study is to analyze the efficacy of intraocular injection of plasma rich in growth factors (PRGF) in an AMD mouse model induced by intraperitoneal administration of sodium iodate. *Materials and Methods*: Intravitreal application of PRGF (experimental group) and saline (control group) was performed immediately after intraperitoneal injection of sodium iodate. Retinographies were performed at 2 and 7 days after treatment administration. The eyes were retrieved for histological and immunohistological analysis. Statistical analysis was performed to compare the outcomes between the study groups. *Results*: In comparison to saline solution, PRGF significantly decreased the depigmentation of the RPE, showing a more reddened retina. PRGF intravitreal treatment significantly reduced the glial fibrillary acidic protein (GFAP) stained processes, suggesting a significant reduction in the risk of scar formation. Moreover, the myofibroblast invasion into the RPE cell layer was significantly reduced in the PRGF-treated group of mice. There was a tendency for better preservation of the photoreceptors in the PRGF group. *Conclusions*: Within the limitations of this study, intravitreal injection of PRGF provided significant protection against the degeneration of the photoreceptors and the RPE induced by the systemic administration of NaIO_3_.

## 1. Introduction

Age-related macular degeneration (AMD) is a chronic and progressive disease of the central retina (termed the macula) that is influenced by genetic and environmental factors. The main nongenetic risk factors are advanced age and smoking. AMD is characterized by the loss of central visual acuity, with a spectrum of clinical phenotypes depending on the changes in the macula. AMD is the leading cause of low vision and legal blindness in adults in developed countries, affecting millions of people worldwide [1,2]. Due to the aging population, the number of people with AMD is expected to increase exponentially over the next decade. Globally, AMD affects approximately 196 million people and is projected to affect 288 million by 2040 [3]. The prevalence of this degenerative eye disease rises from 4.2% in individuals aged 45–49 years to 27.2% in those aged 80–85 years [4,5].

The outer retina, including the retinal pigment epithelium (RPE), Bruch’s membrane, choriocapillaris, and underlying choroid, is primarily affected by AMD. The RPE provides essential retinal homeostatic functions such as nutrient absorption, phagocytosis, and electrolyte balance, while the choriocapillaris and choroid contain an enriched vascular network that nourishes the retinal outer layers [6]. The Bruch membrane facilitates interactions between the RPE and choriocapillaris [7]. In AMD, RPE dysfunction and atrophy occur throughout the progression of the disease, compromising photoreceptor health and the phototransduction process. Additionally, the Bruch membrane plays a pivotal role in the development of neovascular lesions associated with AMD.

Clinical examination of human retinas can reveal distinct hallmarks of AMD that can be broadly divided into early/intermediate and advanced stages. Early/intermediate AMD is the most common and least severe form, characterized by pigmentary abnormalities in the macula and accumulation of extracellular aggregates (called drusen). Late AMD is usually subdivided into dry (or non-exudative) and wet (or exudative) [8]. Wet AMD (~10% of cases) involves choroidal neovascularization into the subretinal space and RPE, eventually leading to retinal hemorrhage, exudation, and severe vision loss. Dry AMD accounts for ~90% of cases, and the advanced stage is defined as geographic atrophy. Atrophic alterations are characterized by progressive and irreversible loss of photoreceptors, RPE, Bruch membrane, and the underlying choriocapillary vascular network [7]. Wet AMD can be successfully treated using vascular endothelial growth factor (VEGF) inhibitors; however, most treated patients have visual impairment due to severe alterations in the retinal tissue, such as fibrosis and/or atrophy [9]. Unlike wet AMD, dry AMD currently has no effective treatment [9]. However, there is evidence showing the safety and effectiveness of rheopheresis of blood plasma in the treatment of dry AMD [10].

AMD places a significant socio-economic burden on healthcare systems, families, and caregivers due to its chronicity and tendency to progress. It limits independence, increases the risk of accidents, impacts emotional well-being (anxiety, depression), and hinders social interaction [11]. All these facts make it necessary to identify new therapeutic targets for this serious disease [7,9,12].

Although the pathogenic mechanisms of the disease are not fully understood, several hypotheses have been proposed, including RPE dysfunction, inflammation, and oxidative stress (the production and accumulation of reactive oxygen species (ROS). RPE cells are rich in mitochondria, and the presence of ROS can disrupt their function, reduce ATP production, and lead to oxidative stress and cell death [13,14]. ROS production can be increased by several factors, including the aging process, smoking, and obesity, all of which contribute to the detriment of RPE cells [15]. To address this situation, researchers have been trying to find a solution to reduce this damage through the use of antioxidants and anti-inflammatory drugs.

Plasma rich in growth factors (PRGF) is a type of platelet rich plasma obtained from the patient’s own blood [16]. It has been shown that PRGF can act as a protector against fibrosis, inflammation, and oxidative stress, mainly due to the wide range of proteins and growth factors that are released into the milieu after platelet activation [17,18,19]. Its beneficial effect has been demonstrated for the treatment of several ocular surface disorders, such as dry eye, persistent epithelial defects (PED), or corneal ulcers, and even in some retinal pathologies like macular holes [20]. In addition, several in vitro and in vivo studies have shown that PRGF exerts a cytoprotective effect on RPE cells against oxidative damage [17,21,22,23]. All of this suggests that PRGF could mitigate retinal tissue damage occurring during AMD progression by providing a protective effect against oxidative stress, highlighting its translatability to human clinical trials and offering a potential innovative therapeutic approach for patients with AMD.

While PRGF’s regenerative potential is well-documented in other fields, its specific mechanisms, efficacy, and safety in treating AMD remain underexplored. Systemic administration of sodium iodate (NaIO_3_) has been widely used as an AMD model that induces a focal atrophic area mimicking human geographic atrophy [24,25,26]. The purpose of the present study was to analyze the efficacy of intraocular injection of PRGF in an AMD mouse model induced by intraperitoneal administration of sodium iodate. The study would provide insights into its therapeutic applicability and bridge the knowledge gap, setting the stage for further translational research and clinical trials.

## 2. Materials and Methods

The study was conducted following the European Community guidelines for ethical animal care and use of laboratory animals (Directive 2010/63/UE) and was approved by the University of Navarra Animal Research Review Committee (47E2022). Male and female C57BL/6J mice (Charles River, Wilmington, MA, USA) between 8 and 12 weeks old were used for this study. The mice were raised under controlled lighting conditions (12:12 light–dark) with free access to a standard diet and tap water.

### 2.1. Experimental AMD Animal Model

Sodium iodate (NaIO_3_) was injected intraperitoneally into mice that were anesthetized by an intramuscular injection of a mixture of ketamine (80 mg/kg; Daiich-Sankyo, Tokyo, Japan) and xylazine (6 mg/kg; Bayer, Health Care, Osaka, Japan). NaIO_3_ (Sigma-Aldrich Corp., St. Louis, MO, USA) was dissolved in phosphate-buffered saline (PBS), and the mice were injected with 20 mg/kg or 40 mg/kg of NaIO_3_. Control mice were injected with the same volume of PBS.

### 2.2. Study Treatments

The experimental treatment was PRGF supernatant, and the control treatment was saline solution. These treatments were injected intravitreally immediately after the intraperitoneal injections of sodium iodate or PBS solutions.

For the preparation of PRGF supernatant, six C57BL/6 mice (3 female and 3 male) were used. The animals were anesthetized prior to the euthanasia process using a CO2 gradient. Blood was then drawn from the individual mice by intracardiac puncture and placed into 2.7 mL tubes containing 3.2% (*w*/*v*) sodium citrate. An aliquot of the pooled peripheral blood was collected for analysis of platelet and leukocyte concentrations. The blood was then centrifuged at 400× *g* for 8 min, and the whole plasma column above the leukocyte coat was collected. An aliquot of pooled plasma was collected for platelet and leukocyte concentration analysis. The total volume of plasma was then activated with calcium chloride and incubated for 1 h at 37 °C. Finally, the supernatant was collected and filtered through a 0.22 μm filter to obtain PRGF supernatant (PRGF).

### 2.3. Macroscopic Analysis

Animals were initially anesthetized with gas anesthesia isoflurane (Merial; Animal Health Ltd., Essex, UK). Then, ketamine (75 mg/kg; Imalgene 1000; Merial Laboratories, Barcelona, Spain) and xylazine (10 mg/kg; Xilagesic 2%; Calier Laboratories, Barcelona, Spain) were used for subsequent anesthesia. Eyes were dilated with a mixture (1:4) of phenylephrine (7.8 mg/mL; Alcon Cusí, Barcelona, Spain) and tropicamide (3 mg/mL; Alcon Cusí, Barcelona, Spain) eye drops. Retinography images were captured using a digital laser system (Micron IV, Phoenix Research Laboratories, Bend, OR, USA) on days 2 and 7 after treatment application.

### 2.4. Tissue Collection and Histopathological Analysis

Mice were anesthetized prior to the euthanasia process using a CO_2_ gradient. The eyes were enucleated using specialized surgical material and were embedded in Davidson for fixation and were subsequently dehydrated in ethanol and embedded in paraffin. Paraffin samples were cut into 4 µm retinal sections. In order to obtain an overview of the whole globe, slices were collected from several sections of the eye (one section every thirty slices, from peripheral to central areas) by taking three slices in each slide. Conventional hematoxylin-eosin (H&E) staining was performed on retinal sections from all groups to observe histologic and structural changes, and images were captured under brightfield microscopy (Axio Imager M1, Zeiss, Oberkochen, Germany). The total thickness of retinal layers, swelling, vacuolization, and the presence of unusual cells in all retinal layers were quantified and compared among the different treatment groups.

### 2.5. Immunohistochemical Analysis

Retinal sections were deparaffinized and hydrated, and the immunohistochemical procedures were performed as described below. Endogenous peroxidase activity was blocked with 3% H_2_O_2_ for 10 min and washed with distilled water for 5 min. If antigen retrieval was necessary (Table 1), sections were placed in 0.01 M citrate buffer (pH 6.0) and heated in a microwave oven for 15 min at medium power (350 W). The sections were then cooled for 10 min at room temperature and placed under tap water for 5 min. Background blocking was performed with 10% fetal bovine serum (FBS) in PBS for 30 min at room temperature before incubation with specific antiserum. The retinal sections were then exposed for 1 h at room temperature to primary antibodies (Annex 1). After washing with PBS, tissues were incubated for 30 min with the appropriate ready-to-use biotinylated secondary antibody, goat anti-rabbit or goat anti-mouse Ig (Abcam, Cambridge, UK), and washed twice with PBS for 5 min. After that, the slices were incubated with ready-to-use avidin-peroxidase complex (Abcam, Cambridge, UK) for 30 min at room temperature. The peroxidase activity was revealed using the Vector VIP kit (Vector Laboratories, Newark, CA, USA) according to the manufacturer’s instructions. The color reaction was stopped by a wash in PBS. The sections were counterstained with hematoxylin, dehydrated, and mounted. Finally, sections were examined under a Leica DM LB brightfield microscope equipped with a digital image capture system (Leica Microsystems). Three images of three different retinal areas were captured from each of the three slices of each slide, and the different images were analyzed using Fiji (ImageJ, National Institutes of Health, Bethesda, MD, USA).

### 2.6. Statistical Analysis

Statistical analyses were carried out with the SPSS software package (v.15.0, SPSS Inc., Chicago, IL, USA). To analyze the differences between the experimental group and the control group, ANOVA or Kruskal–Wallis tests were performed. A *p* < 0.05 was considered statistically significant.

## 3. Results

**Figure 1:**
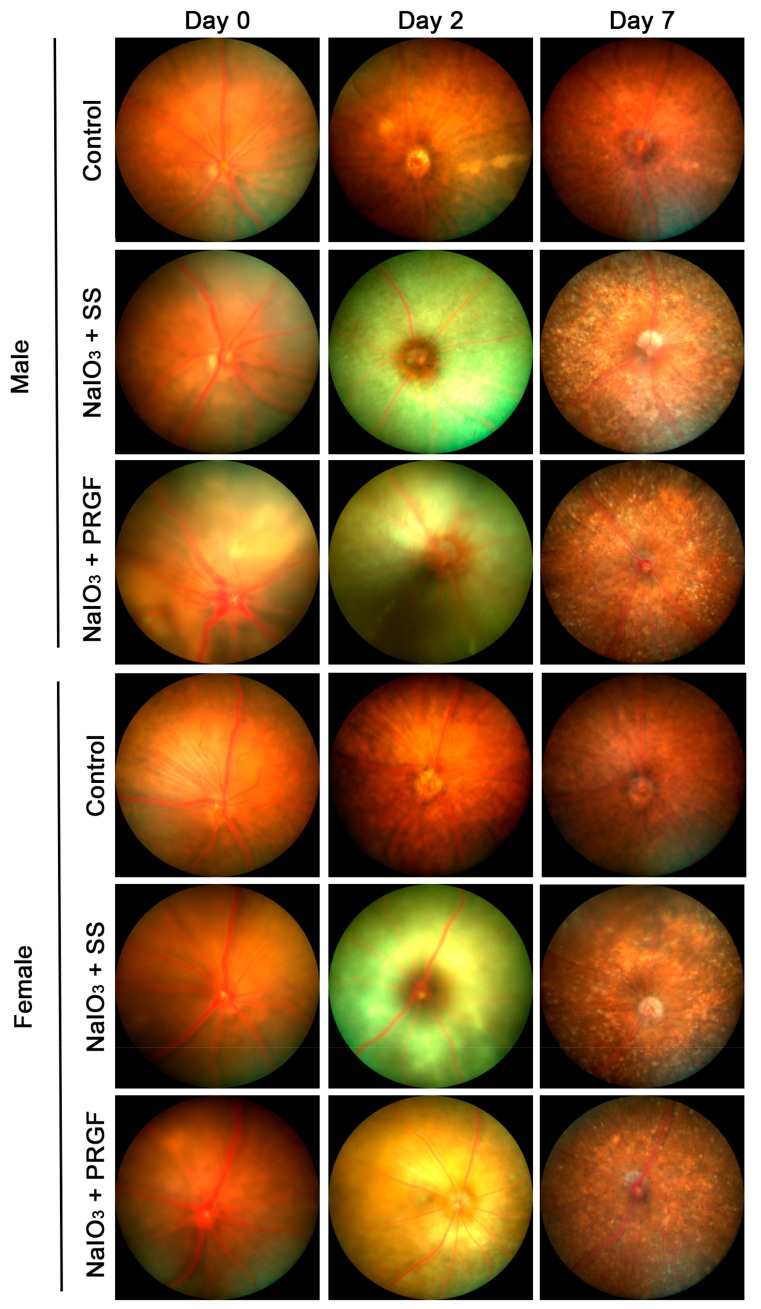
Representative macroscopic images of retinas on the same day of treatment (t0) and after 2 and 7 days of treatment administration from female and male mice of the different groups (control, NaIO_3_ -+ SS, and NaIO_3_ + PRGF). The control group showed the typical retinal redness throughout the study period, while the disease group (NaIO_3_ -+ SS) showed severe depigmentation of the retinal fundus at 2 days after intraperitoneal injection, which recovered at 7 days after treatment, but not to levels similar to the control group. The group of mice treated with PRGF (NaIO_3_ + PRGF) showed significantly less retinal depigmentation than the disease group on day 2, with a more reddened retina. At day 7, retinal depigmentation was significantly reduced, showing a white patch throughout the retina, but this patch was smaller than in the disease group (NaIO_3_ + SS). Macroscopic images show the different effects of NaIO_3_ depending on the sex of the mice, showing a significant reduction effect on the retina of female mice compared to the retina of male mice.

All animals survived the experimental period. Figure 1 illustrates the in vivo monitoring performed on mice belonging to each treatment group, segmented by gender (males and females). The control group showed no alteration in the retina or in the RPE cells. However, retinograms of the disease group (NaIO_3_ + SS), injected with saline intravitreally at the same time as sodium iodate injection, showed acute lesions with severe depigmentation of the RPE at day 2 after intraperitoneal injection. After 1 week, this damage was reduced, and depigmentation was partial, with strong white stippling throughout the retina (Figure 1). In the case of the PRGF-treated group (NaIO_3_ + PRGF), depigmentation of the RPE was significantly lower than in the disease group at day 2, showing a more reddened retina. At day 7, retinal depigmentation was significantly reduced, showing white stippling throughout the retina; however, this stippling was less than in the disease group (NaIO_3_ + SS) (Figure 1). Regarding gender, female mice showed a lower effect after treatment with NaIO_3_ in comparison to male mice at both days of follow-up (2 and 7 days). This fact led to a reduction in the possible effects of the PRGF treatment on the retinal lesions produced by the injection of NaIO_3_ in the female mice. Despite this, a reduction of retinal depigmentation could be observed in the PRGF-treated group (NaIO_3_ + PRGF) compared to the disease group (NaIO_3_ + SS) in female mice at days 2 and 7 of follow-up (Figure 1).

**Figure 2:**
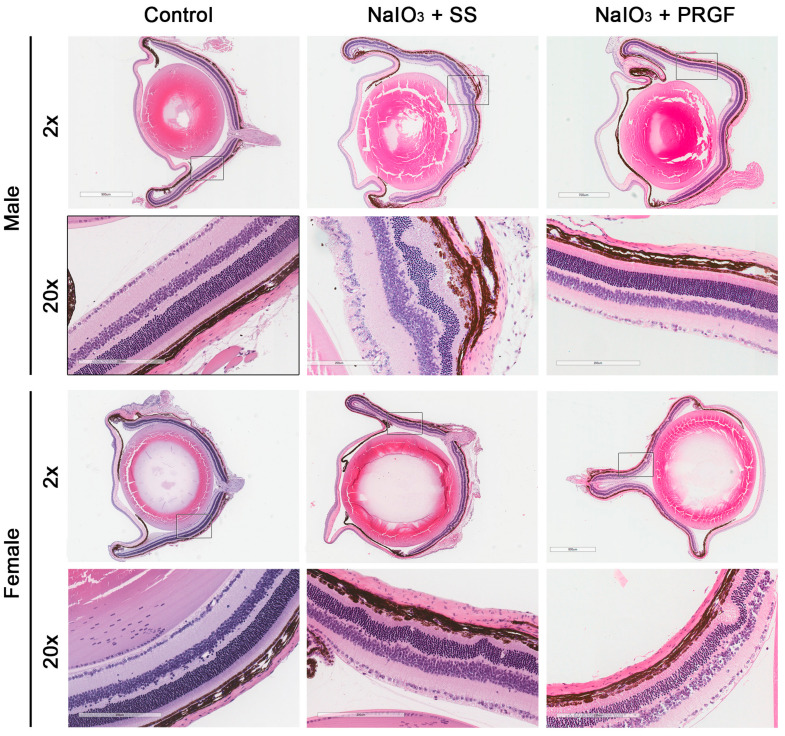
Representative histological images of female and male mice from the different treatment groups (control, NaIO_3_ -+ SS, and NaIO_3_ + PRGF) at two different magnifications (2× and 20×). Images at 20× magnification correspond to the black boxed area drawn in the lower magnification (2×) upper image. The histological images show remarkable retinal disorganization in the disease group of mice compared to the control group and the PRGF group, while the retinas of the PRGF-treated mice showed similar histological morphology to the control group.

The control group showed a normal structure with no alterations in the retina, RPE, and choroid, both in the central areas of the retina, close to the optic nerve, and in the more peripheral retinal areas (Figure 2).

**Figure 3:**
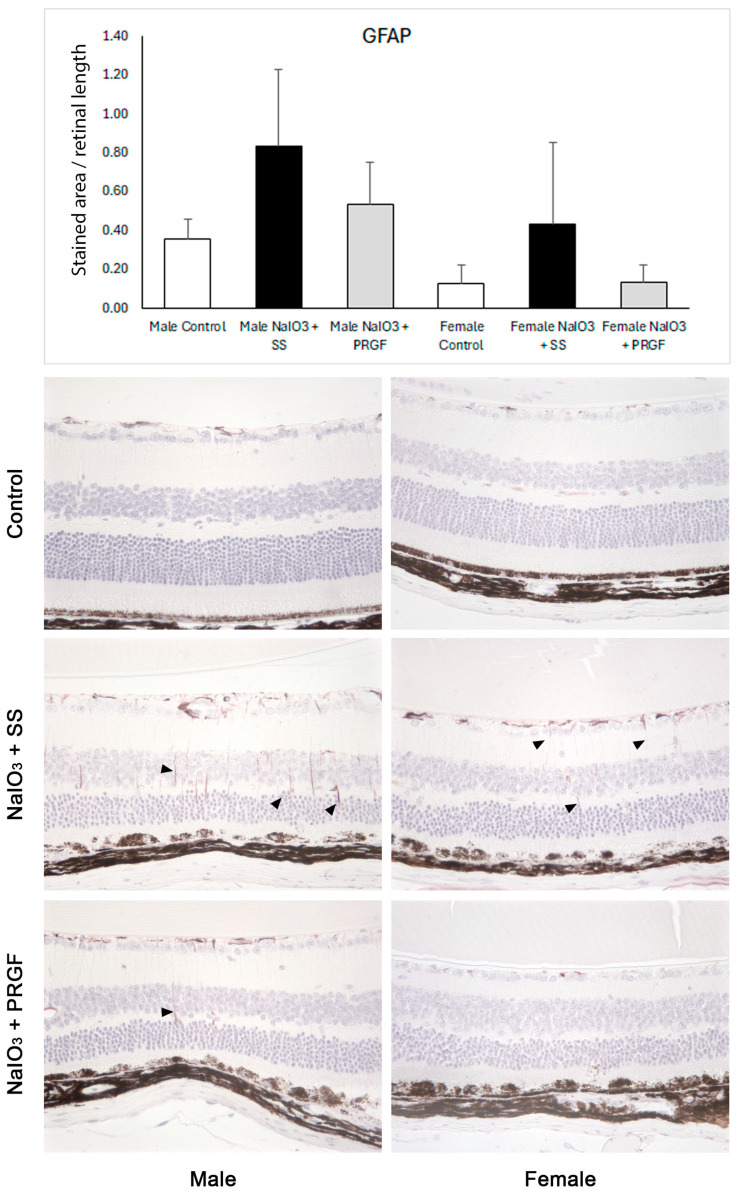
Immunohistochemical analysis of GFAP protein expression. The results showed that GFAP expression increased after intraperitoneal administration of NaIO_3_ compared with the control group. On the other hand, intravitreal administration of PRGF reduced GFAP expression. However, no significant differences were observed between the different groups. Representative GFAP immunohistochemical images show an increased number of GFAP-positive processes throughout the retina of mice in the disease group (black arrowheads) compared with the control and PRGF groups. Noteworthy differences in GFAP protein expression were observed in relation to the mice’s genders.

In the disease group (NaIO_3_ + SS), both male and female mice showed retinal alterations, mainly affecting the RPE cells, and secondarily the rest of the retinal layers (Figure 2). In general, and in correlation with the results observed in the macroscopic evaluation, the damage caused by sodium iodate was less severe in females than in males. On the other hand, when PRGF is injected intravitreally (group NaIO_3_ + PRGF), a very clear reduction of alterations and lesions is observed when compared to the disease group, showing, in some cases, a retinal morphology similar to the control group (Figure 2). In this regard, immunohistochemistry for glial fibrillary acidic protein (GFAP) was performed to evaluate retinal cell damage, activation of glial cell response, and scar formation (Figure 3). Immunohistochemical analysis of GFAP protein expression in the ganglion cell layer (GCL) showed that GFAP expression increased after intraperitoneal administration of NaIO_3_ compared to the control group. In addition, GFAP expression in the GCL decreased after PRGF treatment (NaIO_3_ + PRGF), although no significant differences were found between the different groups (Figure 3). On the other hand, intraperitoneal administration of NaIO_3_ increased GFAP-stained processes extending through the retinal layers (see Figure 3, NaIO_3_+ SS group), compared with the control group, indicating an increase in glial activity, with a significant increase in the risk of scar formation. Nevertheless, PRGF intravitreal treatment significantly reduced the GFAP-stained processes, suggesting a significant reduction in the risk of scar formation. In relation to gender, female mice showed reduced GFAP expression compared to males in all study groups (Control, NaIO_3_ + SS, and NaIO_3_ + PRGF), suggesting again a differential sex-dependent involvement of induced damage and, in this case, also a differential expression of GFAP.

**Figure 4:**
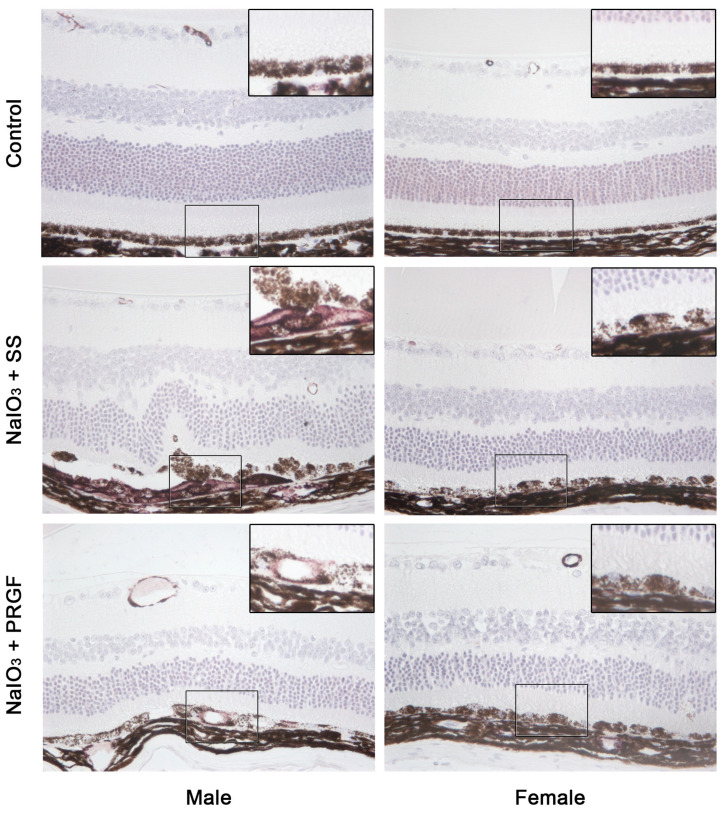
Smooth muscle alpha actin (SMA) expression in the mice retina of the different treatment groups. The squared area in the retina in each image has been enlarged in the upper left corner of the same image. SMA expression was significantly increased in the retinal choriocapillary area in mice in the disease group compared to the control group. PRGF treatment reduced SMA expression in the disease group. On the other hand, a clear difference in SMA expression was observed between female and male retinas.

The most important findings observed during histological analysis were hypopigmentation and hyperpigmentation of various cells located in the RPE cell layer in the disease group (NaIO_3_ + SS) with respect to the control group (see Figure 2). These cell types were mainly located in the central areas of the retina. In addition, giant hyperpigmented cells were observed that, in some cases, had migrated to the inner retina, demonstrating that the tight junctions of the RPE cells had been disrupted or even disappeared. PRGF treatment reduced both hypo- and hyperpigmented cells compared to the disease group (Figure 2). Disruption of the RPE layer increases the likelihood of retinal invasion by vessels and myofibroblasts (positive for alpha smooth muscle (SMA) marker) from subretinal tissues, which increases the risk of scar formation. Microscopic evaluation of immunohistochemistry performed for the detection of SMA showed a significant increase of SMA-positive cells invading the RPE cell layer in mice belonging to the NaIO_3_ + SS group compared with the PRGF-treated group of mice (Figure 4). These cells were mainly observed in the retinal areas where the disruption of the RPE layer was more evident. The control group showed no SMA staining in the RPE or retinal layers, except for staining of vessels located in the innermost retinal layers. In addition, female retinas treated with NaIO_3_ showed a lower presence of SMA-positive cells compared to male retinas, corroborating again a differential effect of NaIO_3_ action between males and females in the induction of retinal damage.

**Figure 5:**
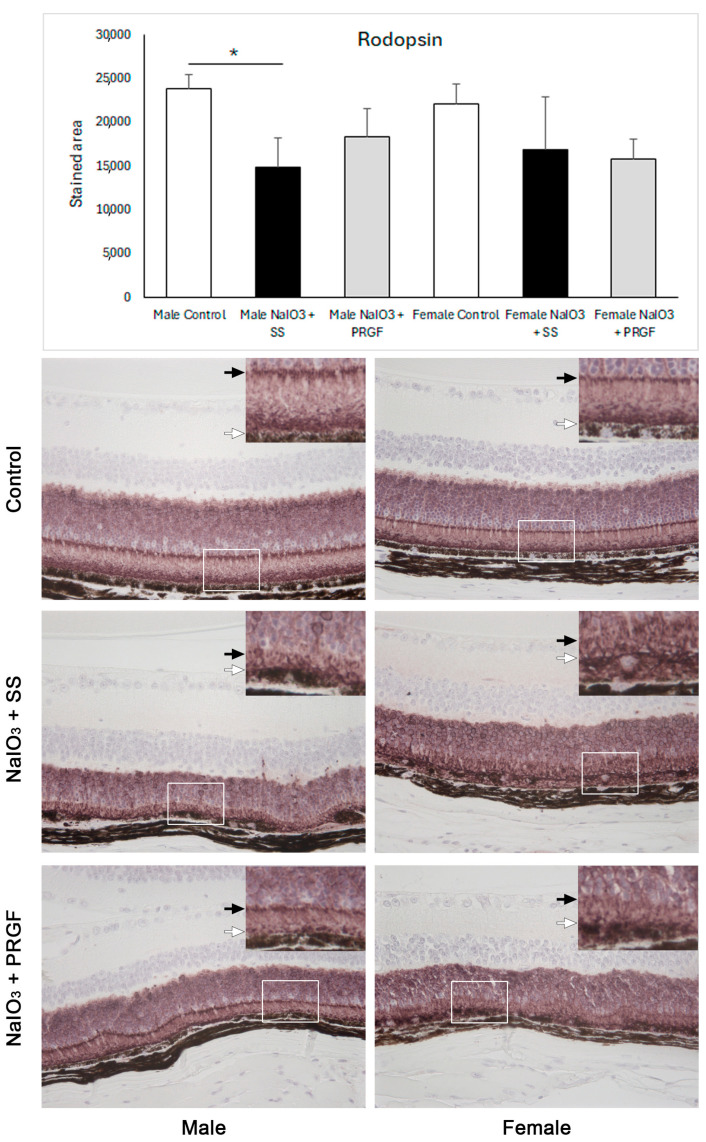
Immunohistochemical analysis of rhodopsin in the retina of treated mice. The results showed that rhodopsin was significantly reduced in the retina of mice in which NaIO_3_ was administered intraperitoneally compared with the control group. On the other hand, PRGF increased rhodopsin expression with respect to the disease group, though without reaching statistical significance. Rhodopsin immunohistochemical imaging shows that, although the rhodopsin-positive area was markedly higher in the PRGF group than in the disease group, no significant differences were observed between them. The image in the upper left corner of each image corresponds to the magnification of the white-boxed retinal area of the same image. The enlarged image shows that the length of the photoreceptor outer segments, delimited between the black arrow and the white arrow, is significantly shorter in the retina of the disease group compared with the control group and the PRGF group. *: statistically significant (*p* < 0.05).

Changes in RPE cells due to the action of iodate in the disease group induced a marked decrease in the thickness of photoreceptor outer segments, suggesting a loss of photoreceptor outer segments with a clear apoptotic phenotype in the nucleus compared with the control and PRGF-treated groups (Figure 2). In some areas, there was a loss of photoreceptor layers and involvement of the inner retinal layers. Immunostaining for rhodopsin (photoreceptor marker) showed a significant reduction in the disease group (NaIO_3_ + SS) compared to the control group in male mice (Figure 5). However, this reduction was attenuated after treatment with PRGF (NaIO_3_ + PRGF), though without reaching significant differences. In the case of female mice, although there was a trend toward a decrease in rhodopsin immunostaining in the iodate-treated mice in both the diseased and PRGF-treated groups compared to the control group, no significant differences were observed between any group (Figure 5). However, the loss of photoreceptor outer segments was corroborated with the rhodopsin immunostaining, showing an outstanding reduction of the photoreceptor outer segments in the mice in the disease group (NaIO_3_ + SS) in both genders compared to the control group and the group treated with PRGF (NaIO_3_ + PRGF). Although a slight reduction in photoreceptor outer segments was also observed in mice in the PRGF-treated group compared with the control group, the preservation of these segments was markedly superior in the PRGF-treated mice compared with those in the disease group (Figure 5).

## 4. Discussion

The increase in life expectancy has led to a rise in the incidence of age-related diseases [27], including age-related macular degeneration (AMD). Dry AMD is characterized by the accumulation of drusen, yellow deposits beneath the retina, which leads to the thinning and atrophy of the retinal pigment epithelium and photoreceptors [28,29]. Additionally, dry AMD involves the progressive degeneration of retinal cells, leading to substantial vision loss [28,30].

Currently, there is no effective treatment for dry AMD, and the few therapeutic options are largely limited to interventions aimed at slowing disease progression. Unlike wet AMD, which can be treated with anti-VEGF therapies to inhibit abnormal blood vessel growth, dry AMD lacks such targeted treatments [9]. Research efforts are ongoing to develop novel therapies that can address the mechanisms underlying dry AMD, such as complement pathway inhibitors, neuroprotective agents, and anti-oxidative therapies [31]. In this regard, several neuroprotective factors, such as glia-derived neurotrophic factor (GDNF), nerve growth factor (NGF), brain-derived growth factor (BDNF), neurotrophin 3 (NT-3), and neurotrophin 4 (NT-4), have emerged as alternative treatments to promote neuronal survival, regeneration, and plasticity in different retinal diseases [32]. In addition, the response to oxidative stress and the progression of dry AMD involve several growth factors. On the one hand, there are factors such as vascular endothelial growth factor (VEGF) and transforming growth factor-beta (TGF-β), which are involved in inflammation and the response to oxidative stress, and whose increase may exacerbate oxidative damage and inflammatory processes in the retina. On the other hand, several growth factors, such as PEDFs, FGFs, and PDGFs, have a clear role in cell survival and proliferation and have potent antioxidant and anti-inflammatory effects [33,34]. These findings suggest that growth factor therapies could restore retinal homeostasis, slowing AMD progression and preserving vision.

Different studies have shown that plasma rich in growth factors (PRGF) has a broad content of growth factors involved in tissue regeneration, including neuroprotective factors such as NGF, FGF, or PDGF [35,36]. In addition, recent results have shown that PRGF can exert a cytoprotective effect on RPE cells that have been exposed to an oxidative environment, restoring the counterbalance between PEDF and VEGF [17]. These results suggest that PRGF and its wide growth factor content may act to counteract the effects of AMD on the RPE and retinal tissues.

In this study, a mouse model that mimics the loss of RPE and photoreceptors observed in retinal degenerative diseases, such as AMD was performed using an intraperitoneal injection of NaIO_3_ [37,38]. As in a fundus image in the severe phase of AMD progression, mice treated intraperitoneally with NaIO_3_ showed atrophic hypopigmented retinal lesions indicating progressive loss of the RPE, photoreceptors, and underlying choriocapillaris [39]. The intravitreal application of PRGF significantly reduced the RPE loss due to the NaIO_3_ injection. The retina appeared more reddened at days 2 and 7 in the PRGF group. Several studies suggest that the loss of the RPE leads to the atrophy of the choroid and its choriocapillaris, which may result in scar formation [26]. Scar tissue is mainly composed of myofibroblastic cells expressing different types of contractile filaments, such as alpha-smooth muscle actin (α-SMA) [40]. Myofibroblastic cells play a critical role in AMD pathogenesis. They participate in the fibrotic changes observed in the disease, contributing to fibrotic scar formation in the macula. Their presence contributes to the development of subretinal fibrosis and is associated with the progression of AMD, especially in advanced stages [41,42]. In addition, this choriocapillaris degeneration and subsequent scarring have also been observed in the AMD model obtained after intraperitoneal injection of NaIO_3_ [43]. The present study shows that intraocular PRGF treatment reduced SMA immunostaining in the retinal choriocapillaris area related to the presence of myofibroblasts compared with the disease group. These results suggest that PRGF may protect RPE cells, reducing their loss and, therefore, its consequent choriocapillary degeneration. 

During the pathogenesis of AMD, Müller glial activation, remodeling, and migration occur specifically in areas of RPE atrophy, photoreceptor loss, and choroidal neovascularization lesions. These GFAP- and vimentin-positive glial cells (markers of Müller glial activation or gliosis) are observed directly overlying areas of RPE degeneration, suggesting a strong association between activated Müller glial migration and RPE pathology in AMD [44]. This activation of Müller glial cells has also been observed in retinal tissues from animal models of AMD treated with sodium iodate, showing very marked GFAP immunostaining, with radial processes of Müller cells through the neuroretinal layers [45]. The results obtained in this study showed that intraocular injection of PRGF significantly reduced the number of processes immunostained for GFAP. These results suggest that PRGF reduces Müller cell activation, probably due to reduced RPE cell damage and photoreceptor loss. Similar results were observed in a previous study in which PRGF administration significantly reduced GFAP expression in rat retinas exposed to blue light as an oxidative AMD model [22].

The progression of AMD to more advanced stages leads to geographic atrophy, which is characterized by a thinning of the neuroretinal layers and results in severe visual impairment [8]. NaIO_3_ intraperitoneal injection leads to the thinning of the whole retinal layers, affecting not only the RPE cells, but also photoreceptors, retinal ganglion cells, and bipolar cells [26]. The results observed in the present study show that intraocular injection of PRGF reduced retinal tissue thinning compared with the disease group. These results became more notable when rhodopsin immunostaining was used to detect photoreceptors; however, histomorphometric analysis of rhodopsin-positive retinal areas showed no significant differences between the PRGF and disease groups. Similar results have been observed by Liu et al., where they also observed a thinning of the retinal layers after NaIO_3_ treatment, but they did not detect significant differences between the treated and control groups after histomorphometric analysis [46]. On the other hand, several studies have shown an apparent shortening of the outer segments of the rods and cones in NaIO_3_-injected rats compared to the control group [44]. In the present study, rhodopsin immunostaining revealed a marked reduction of photoreceptor outer segments in mice treated intraperitoneally with NaIO_3_; however, intraocular treatment with PRGF showed remarkable protection, with longer photoreceptor outer segment lengths more similar to those of the uninjured control group.

It is important to highlight that the results of the present study indicated the impact of gender on the experimental model of AMD, showing a significant reduction of retinal damage in female mice, even compromising the effect that PRGF could exert. These results are in contrast to those of Kiuchi et al., where no sex differences were observed in response to NaIO_3_ [45].

Although this is a preliminary study and further studies are needed, this study highlights important findings to be taken into consideration: sex is an influencing factor in both model generation and treatment efficacy, the model represents the acute injury to the retina (a limitation of the model), and the feasibility of using allogenic blood to produce PRGF eye drops.

## 5. Conclusions

Age-related macular degeneration (AMD) is a leading cause of vision loss, primarily affecting the retinal pigment epithelium (RPE) and photoreceptors, critical components for vision. The NaIO_3_-induced retinal degeneration model is commonly used to study AMD-like damage. Plasma rich in growth factors (PRGF) has been proposed as a potential therapeutic due to its regenerative properties. In this study, intravitreal injection of PRGF significantly protected against RPE and photoreceptor degeneration induced by systemic NaIO_3_ administration, suggesting its potential as a treatment for retinal diseases like AMD.

## Figures and Tables

**Table 1:** Primary antibodies used in this study.

Antibody	Reference	Species	Dilution	Antigen Retrieval	Company
GFAP	Cat# 3670S	Mouse	1:80	Microwave	Cell Signaling, Danvers, MA, USA
α-SMA	Cat# A2547	Mouse	1:800	-	Sigma-Aldrich, St. Louis, MO, USA
Rhodopsin	Cat# ZBR1157	Rabbit	1:200	-	Sigma-Aldrich, St. Louis, MO, USA

## Data Availability

The datasets used and/or analyzed during the current study are available from the corresponding author on reasonable request.
